# Arterial spin labeling of nasopharyngeal carcinoma shows early therapy response

**DOI:** 10.1186/s13244-022-01248-x

**Published:** 2022-07-07

**Authors:** Jun Liu, Juan Zhu, Yaxian Wang, Fei Wang, Hualin Yang, Nan Wang, Qingyun Chu, Qing Yang

**Affiliations:** 1 Department of Medical Imaging, Anqing Hospital Affiliated to Anhui Medical University, No352, Renmin Road, Yingjiang District, Anqing, 246003 Anhui China; 2 Department of Medical Oncology, Anqing Hospital Affiliated to Anhui Medical University, No352, Renmin Road, Yingjiang District, Anqing, 246003 Anhui China

**Keywords:** Arterial spin labeling, Chemoradiotherapy, Nasopharyngeal carcinoma, Magnetic resonance imaging

## Abstract

**Objective:**

This study aimed to determine the value of arterial spin labeling (ASL) perfusion imaging in assessing the early efficacy of chemoradiotherapy for nasopharyngeal carcinoma (NPC).

**Methods:**

Fifty-five patients with locoregionally advanced NPC underwent conventional 3.0-T magnetic resonance imaging (MRI) and ASL before and after chemoradiotherapy (prescribed dose reached 40 Gy). Based on the response evaluation criteria for solid tumors (RECIST 1.1), the patients were divided into the partial response and stable disease groups. MRI re-examination was performed one month after chemoradiotherapy completion, and patients were divided into residual and non-residual groups. We investigated inter-group differences in ASL-based tumor blood flow (TBF) parameters (pre-treatment tumor blood flow, post-treatment tumor blood flow, and changes in tumor blood flow, i.e., Pre-TBF, Post-TBF, ΔTBF), correlation between TBF parameters and tumor atrophy rate, and value of TBF parameters in predicting sensitivity to chemoradiotherapy.

**Results:**

There were differences in Pre-TBF, Post-TBF, and ΔTBF between the partial response and stable disease groups (*p* < 0.01). There were also differences in Pre-TBF and ΔTBF between the residual and non-residual groups (*p* < 0.01). Pre-TBF and ΔTBF were significantly correlated with the tumor atrophy rate; the correlation coefficients were 0.677 and 0.567, respectively (*p* < 0.01). Pre-TBF had high diagnostic efficacies in predicting sensitivity to chemoradiotherapy and residual tumors, with areas under the curve of 0.845 and 0.831, respectively.

**Conclusion:**

ASL permits a noninvasive approach to predicting the early efficacy of chemoradiotherapy for NPC.

## Key points

- ASL may be suitable to assess early chemoradiotherapy efficacy for NPC.
- High Pre-TBF was predictive of tumor sensitivity to chemoradiotherapy and no residual tumor at the end of treatment.
- Pre-TBF and ΔTBF were highly correlated with the tumor atrophy rate.

## Introduction

Nasopharyngeal carcinoma (NPC) is prevalent in Southeast Asia and shows geographical and ethnic variations [1]. For patients with locoregionally advanced (III–IVA) NPC, the main treatment method at present is radiotherapy combined with concurrent adjuvant platinum-based chemotherapy [2]. Currently, the local tumor control rates and overall 5-year survival rates have been increasing, but local metastasis and recurrence still constitute the main causes of treatment failure [3]. Early assessment of tumor response to treatment is of great clinical significance, and it can guide clinicians in timely and rational adjustments of treatment regimens as well as prognostic evaluation of patients.

Tumor microcirculation is a major factor affecting tumor response to treatment [4]. Good tumor perfusion can facilitate the effective delivery of anti-tumor agents to tumor tissues, and the efficacy of chemoradiotherapy can be enhanced by improving tumor perfusion and local hypoxia within tumor tissues [5]. MRI-based techniques have been widely adopted in NPC staging, target definition for radiotherapy, and efficacy evaluation due to their superior soft-tissue contrast resolution and more comprehensive evaluation of intracranial and retropharyngeal space [6]. Moreover, functional MRI parameters capture changes in tumor microcirculation before tumor morphological changes become apparent, and the value of functional MRI techniques in predicting tumor staging and evaluating efficacy has been confirmed. At present, various imaging techniques have been adopted to evaluate the efficacy of chemoradiotherapy for NPC, including diffusion-weighted imaging (DWI) [7], diffusion kurtosis imaging (DKI) [8, 9], dynamic contrast-enhanced magnetic resonance imaging (DCE-MRI) [9–11], and intravoxel incoherent movement diffusion-weighted imaging (IVIM-DWI) [12, 13]. Three-dimensional pseudo-continuous arterial spin labeling (ASL) imaging was primarily used for the assessment of the central nervous system at the early stage [14], and it has been gradually applied to other sites such as the kidney [15] and bone marrow [16]. There have been reports on ASL application to the clinical staging evaluation [11] and differential diagnosis [17] of NPC, but reports on evaluating the early efficacy of chemoradiotherapy for NPC are yet to be published.

Therefore, we performed a retrospective analysis of the relationships between ASL parameters and early efficacy of chemoradiotherapy for NPC and assessed the potential value of these parameters in predicting sensitivity to chemoradiotherapy for NPC.

## Materials and methods

### Patient selection and treatment procedure

**Fig. 1:**
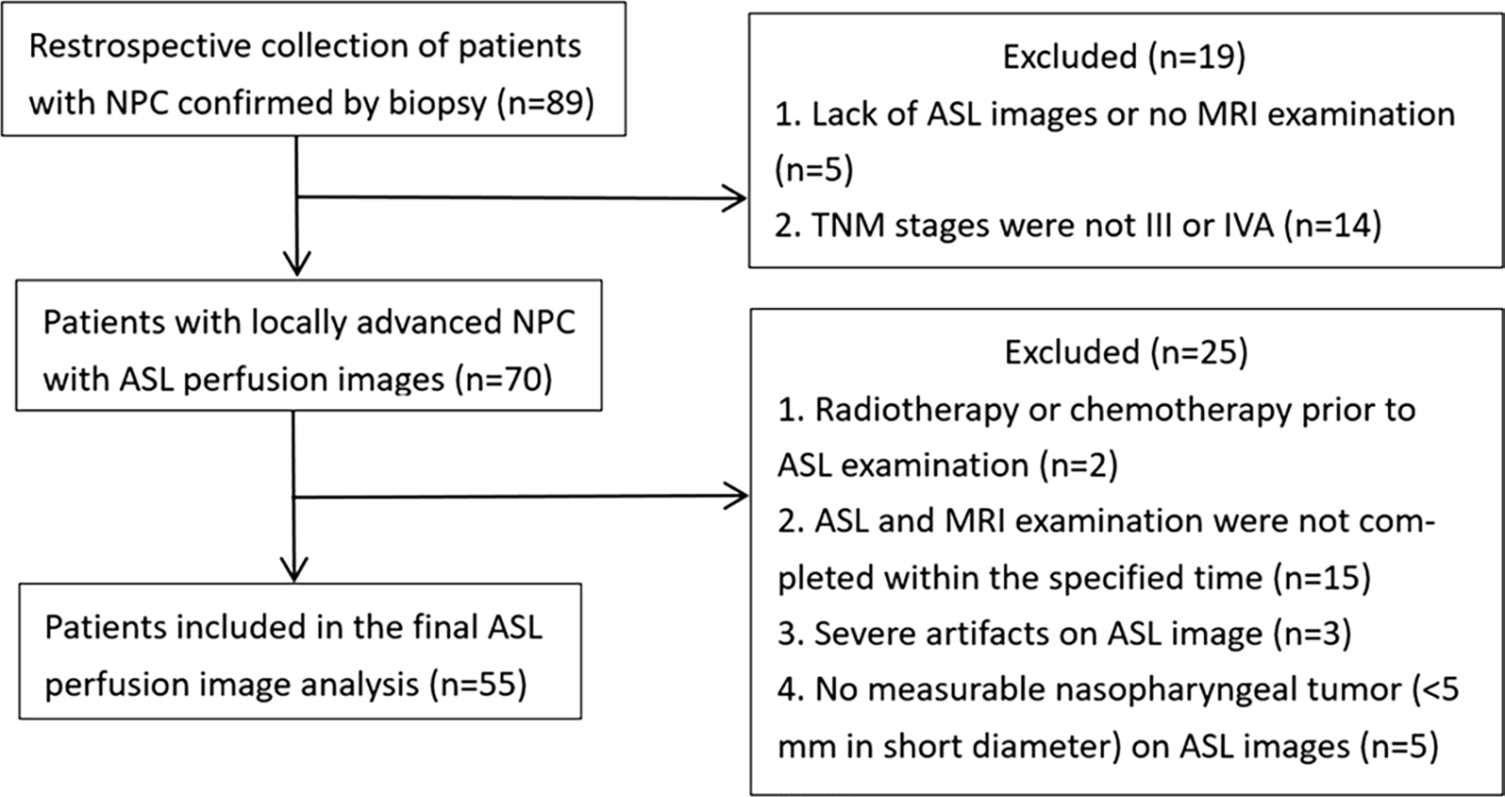
Flow diagram of patient selection

This study was approved by the medical ethics committee of our hospital, and the need for informed consent was waived due to the retrospective nature of this study. We retrospectively assessed 89 NPC patients whose diagnosis was confirmed by pathological examination of nasopharyngeal biopsy specimens from March 2017 to August 2021. The inclusion criteria included the following: patients who did not receive treatment for NPC before MRI scan and had no prior history of head and neck cancer; patients with diagnoses confirmed by pathological examination of biopsy tissues; patients with stage III–IVA NPC (staging based on the 8^th^ Edition of the American Joint Committee on Cancer Staging System for NPC) [18] who received radiotherapy and concurrent cisplatin- or carboplatin-based chemotherapy and completed re-examinations when the prescribed dose reached 40 Gy and at one month after chemoradiotherapy completion. The exclusion criteria were as follows: lack of ASL images or no MRI examination; radiotherapy or chemotherapy before ASL MRI examination; patients with severe artifacts on ASL images or patients whose lesion region of interest (ROI) could not be outlined due to the small sizes of post-treatment tumor lesions. Finally, 55 patients were included for further image analysis (Fig. 1).

The tumor, node, metastasis staging of all patients was co-determined by two radiologists (J.L. and N.W., with 5 and 15 years of experience in head and neck radiology, respectively) based on the findings of head and neck MRI, thoracoabdominal computed tomography, MRI of other body areas, or nuclear medicine exams. The specific treatment regimens for NPC were as follows: a total prescribed dose of 70 Gy delivered to the primary nasopharyngeal lesions in 35 fractions (when calculated using a linear-quadratic formula [19, 20] [Eq. (1)], the total prescribed dose can be converted to a biologically effective dose [BED] of 84 Gy); 66 Gy (BED = 78.45 Gy) to metastatic lymph nodes, 60 Gy (BED = 70.29 Gy) for draining lymph nodes at high risk of containing cancer, and 54 Gy (BED = 62.33 Gy) for draining lymph nodes at low risk of containing cancer; five fractions weekly (one fraction each day) combined with concurrent cisplatin-based chemotherapies. All patients underwent three MR examinations before treatment initiation, when the prescribed dose reached 40 Gy to the primary nasopharyngeal lesions and at one month after chemoradiotherapy completion, with the MR regimen unchanged during the entire process. When the prescribed dose reached 40 Gy, all patients were divided into the partial response (PR) (36 cases) and stable disease (SD) (19 cases) groups based on the universal RECIST 1.1 standard [21]. At one month after chemoradiotherapy completion, the patients were re-divided into the residual (17 cases) and non-residual (38 cases) groups.

1 $${\text{BED}} = D \times \left( {1 + \frac{d}{\alpha /\beta }} \right)$$

BED: Biologically effective dose, in Gy; *D*: Total dose (number of fractions × dose per fraction, in Gy); *d*: Dose per fraction, in Gy; *α*/*β*: Property of irradiated tissue (the value here was 10 Gy).

### MRI acquisition

All MRI scans were performed using a 3.0 T whole-body MR system (Discovery MR 750, GE) with an 8-channel head and neck phased array coil. The routine MRI evaluation included coronal T_2_-iterative decomposition of water and fat with echo asymmetry and least-squares estimation (T_2_-IDEAL) (repetition time [TR] = 2828 ms, echo time [TE] = 68 ms, field of view [FOV] = 28 cm × 28 cm, slice thickness = 5 mm, slice gap = 1 mm, matrix = 288 × 192, number of averages [NEX = 1]); axial T1-weighted imaging (T1WI) (TR = 790 ms, TE = 6.5 ms, FOV = 22 cm × 22 cm, slice thickness = 4 mm, slice gap = 1 mm, matrix = 288 × 256, NEX = 2); and axial T_2_-IDEAL (TR = 3000 ms, TE = 72 ms, FOV = 22 cm × 22 cm, slice thickness = 4 mm, slice gap = 1 mm, matrix = 288 × 192, NEX = 1).

Axial ASL was acquired using 3D fast-spin echo, and the labeling plane was placed 2 cm below the inferior border of the FOV using the following parameters: TR = 5029 ms, TE = 14.6 ms, FOV = 24 cm × 24 cm, slice thickness = 4 mm (with a gap of 0 mm), post-labeling delay (PLD) = 2025 ms, NEX = 3, echo train length (ETL) = 21, number of scanned slices = 36, scan duration = 4 min 52 s, scan range: from the fundus of the frontal sinus to the oral pharynx. Another axial 3D-T1BRAVO series (3D inverse recovery–fast spoiled gradient recalled echo brain volume, BRAVO) was performed with a uniform scan range as that of ASL (TR = 8.2 ms, TE = 3.2 ms, FOV = 24 cm × 24 cm, slice thickness = 1.2 mm, slice gap = 0 mm, matrix = 256 × 256, NEX = 1), which could be fully integrated with ASL, and the fused images were used to plot the ROI during ASL data analysis; finally, gadolinium-DTPA (GD-DTPA) was injected via a median cubital vein at a dose of 0.1 mmol/kg for axial, coronal, and sagittal contrast-enhanced T1WI scanning.

### ASL data analysis

The ASL series were imported to Functool Software (MR 750 scanner post-processing workstation for ASL post-processing software) and integrated with the T1BRAVO series to eventually obtain the tumor blood flow (TBF). TBF was calculated using Eq. (2):

2 $${\text{TBF}} = \frac{{6000 \cdot \lambda \left( {{\text{SI}}_{{{\text{control}}}} - {\text{SI}}_{{{\text{label}}}} } \right) \cdot e^{{\frac{{{\text{PLD}}}}{{T_{{\text{1,blood}}} }}}} }}{{2 \cdot \alpha \cdot T_{{\text{1,blood}}} \cdot {\text{SI}}_{{{\text{PD}}}} \cdot \left( {1 - e^{{ - \frac{\tau }{{T_{{1,{\text{blood}}}} }}}} } \right)}}\left[ {\text{mL/100g/min}} \right]$$

where TBF is the tumor blood flow; *λ* represents the tumor tissue/blood partition coefficient in mL/g; SI_control_ and SI_label_ refer to the time-averaged signal intensities in the control and label images, respectively; *T*_1,blood_ is the longitudinal duration of relaxation of blood in seconds (1650 ms); *α* represents the labeling efficiency (0.85); SI_PD_ is the signal intensity of a proton density-weighted image; *τ* is the label duration; and PLD indicates the post-labeling delay time. A factor of 6000 converts the units from mL/g/s to mL/ (100 g)/min, which is customary in physiological literature.

### Data analysis

The parameters were independently measured by two observers (F.W. and H.L.Y.; 5 and 15 years of experience in head and neck cancer diagnosis, respectively) in a double-blinded state and without knowledge of treatment responses. All data were processed on an ADW 4.6 workstation. First, the maximum primary tumor diameter (MPTD) in the axial, coronal, and sagittal planes was measured separately on contrast-enhanced T1WI images before treatment with T2-IDEAL and T1WI images as references. The maximum value measured in the above three directions was taken as the Pre-MPTD. In the second step, when the prescribed dose reached 40 Gy, an MRI examination with the same scanning scheme was performed, and MPTD measured in the same plane as the same sequence before treatment and taken as Post-MPTD. The third step was to calculate the tumor atrophy rate using Eq. (3):

3 $${\text{Atrophy rate}} = \frac{{{\text{Pre}} - {\text{MPTD}} - {\text{Post}} - {\text{MPTD}}}}{{{\text{Pre}} - {\text{MPTD}}}} \times 100\%$$

where MPTD is the maximum primary tumor diameter.ach observer manually drew three ROIs for each tumor on the fused ASL and axial BRAVO images on the slice showing the largest lesions, neighboring upper slice, and neighboring lower slice to cover the nasopharyngeal tumor lesions as much as possible. Meanwhile, areas of necrosis, air gaps, large vessels, and adjacent anatomic structures had to be avoided when drawing the ROIs. The average measurement over the three ROIs was taken as the TBF value recorded by each observer. Pre-treatment TBF was recorded as Pre-TBF; post-treatment TBF as Post-TBF; and the change in TBF value as ΔTBF. The mean values of the two observers’ measurements were taken as final values for all parameters and the interclass correlation coefficients between the two observers were calculated. The same observer repeated the measurements after one week to calculate the intraclass correlation coefficients.

### Statistical analysis

Quantitative parameters were tested to check whether they follow a normal distribution. Normally distributed quantitative parameters were denoted by $\overline{X}$. ± *S*. SPSS v.23.0 (IBM Corp, Armonk, NY), GraphPad Prism v.8.0 (GraphPad software, San Diego, CA), and MedCalv.15.11.4 (Mariakerke, Belgium) were used for the data analysis. The differences in ASL-derived parameters before and after treatment for NPC were determined using a paired sample t-test; the differences in ASL-derived parameters between the PR and SD groups and between the residual and non-residual groups were analyzed using independent sample t-tests. Pearson's chi-squared test was performed to identify differences between the proportions of residual tumors between the SD and the PR groups at the end of chemoradiotherapy. Pearson correlation analysis was performed to determine the correlation between ASL-derived parameters and the tumor atrophy rate. Receiver operating characteristic (ROC) curves were used to determine the diagnostic efficacy of ASL-derived parameters in predicting sensitivity to chemoradiotherapy and residual tumors. In this study, the intraclass and interclass correlation coefficients with 95% confidence interval were used to define the interobserver and intra-observer consistency, and Bland–Altman plots were used to analyze the interobserver agreement of the parameter measurements. *p* ≤ 0.05 indicates statistical significance.

## Results

### Clinical data and grouping of 55 patients with NPC

Fifty-five patients with locoregionally advanced NPC were finally enrolled in this study: 51 had non-keratinizing carcinoma (41 cases with undifferentiated carcinoma and 10 cases with differentiated carcinoma), and four had keratinizing squamous cell carcinoma. Forty-three patients were male and 12 were female, with a mean age of 55.76 ± 9.26 years. The staging data of the patients are shown in Table 1. Thirty-six patients were allocated to the PR group and 19 to the SD group. Re-examinations one month after chemoradiotherapy completion suggested that 17 patients had residual tumors and 38 did not, with significant differences in the percentage of residual tumor between the SD and PR groups (Table 2).

**Table 1:** Clinical data of patients with NPC

Parameters	Results
Sex (male/female)	43/12
Age (years)	55.76 ± 9.26
Range (years)	38–76
*Pathological type*^*a*^	
Undifferentiated	51
Differentiated	4
*T stage*	
*T*1	0
*T*2	9
*T*3	23
*T*4	23
*N stage*	
*N*1	3
*N*2	32
*N*3	20
*AJCC stage*	
III	26
IVA	29

*NPC*: nasopharyngeal carcinoma; *AJCC* American Committee on Cancer

^a^WHO classification

**Table 2:** Pearson’s chi-squared test analysis

Group	Residual	No-residual	Total
PR	5 (13.9%)	29 (86.1%)	36
SD	12 (63.2%)	7 (36.8%)	19
Total	17	38	55

Pearson’s chi-squared = 14.136, *p* = 0.000

*PR*: the partial response group; *SD* the stable disease group

### Reproducibility of ASL-derived parameters and tumor atrophy rate

**Fig. 2:**
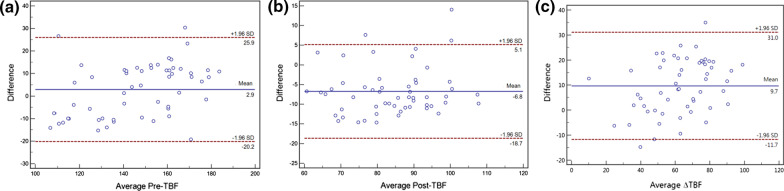
Bland–Altman plots with 95% CI comparing the measurements of the two observers to estimate the interobserver reproducibility for Pre-TBF (**a**), Post-TBF (**b**), and ΔTBF (**c**). The mean differences between the first and the second measurements (*y*-axis) plotted against their averages (*x*-axis). The horizontal lines (blue solid line) are drawn at the mean difference of the two times measurements and the limits of agreement (red dotted lines). The ASL parameter measurements by the two observers showed good agreement

The interclass correlation coefficients (95% CI) indicating interobserver reproducibility for Pre-TBF, Post-TBF, ΔTBF, and tumor atrophy rate were between 0.912 and 0.985, whereas the intraclass correlation coefficients (95% CI) indicating that intra-observer reproducibility fell between 0.894 and 0.994. The correlation coefficients suggested good interobserver and intra-observer reproducibility and agreement for the parameters (Table 3). The Bland–Altman analysis also showed good interobserver reproducibility, which is acceptable in clinical practice (Fig. 2).

**Table 3:** Reproducibility of ASL-derived parameters and tumor atrophy rate

Parameters	ICC	
	Intra (95% CI)	Inter (95% CI)
Pre-TBF	0.912 (0.849, 0.949)	0.924 (0.871, 0.956)
Post-TBF	0.943 (0.902, 0.967)	0.931 (0.882, 0.960)
△TBF	0.894 (0.818, 0.938)	0.912 (0.849, 0.949)
Atrophy rate%	0.994 (0.990, 0.996)	0.985 (0.974, 0.991)

*Pre-TBF* pre-treatment tumor blood flow; *Post-TBF* post-treatment tumor blood flow; *ΔTBF* changes in tumor blood flow; *CI* confidence interval

### ASL-derived parameters in each group and their correlation with tumor atrophy rate

**Fig. 3:**
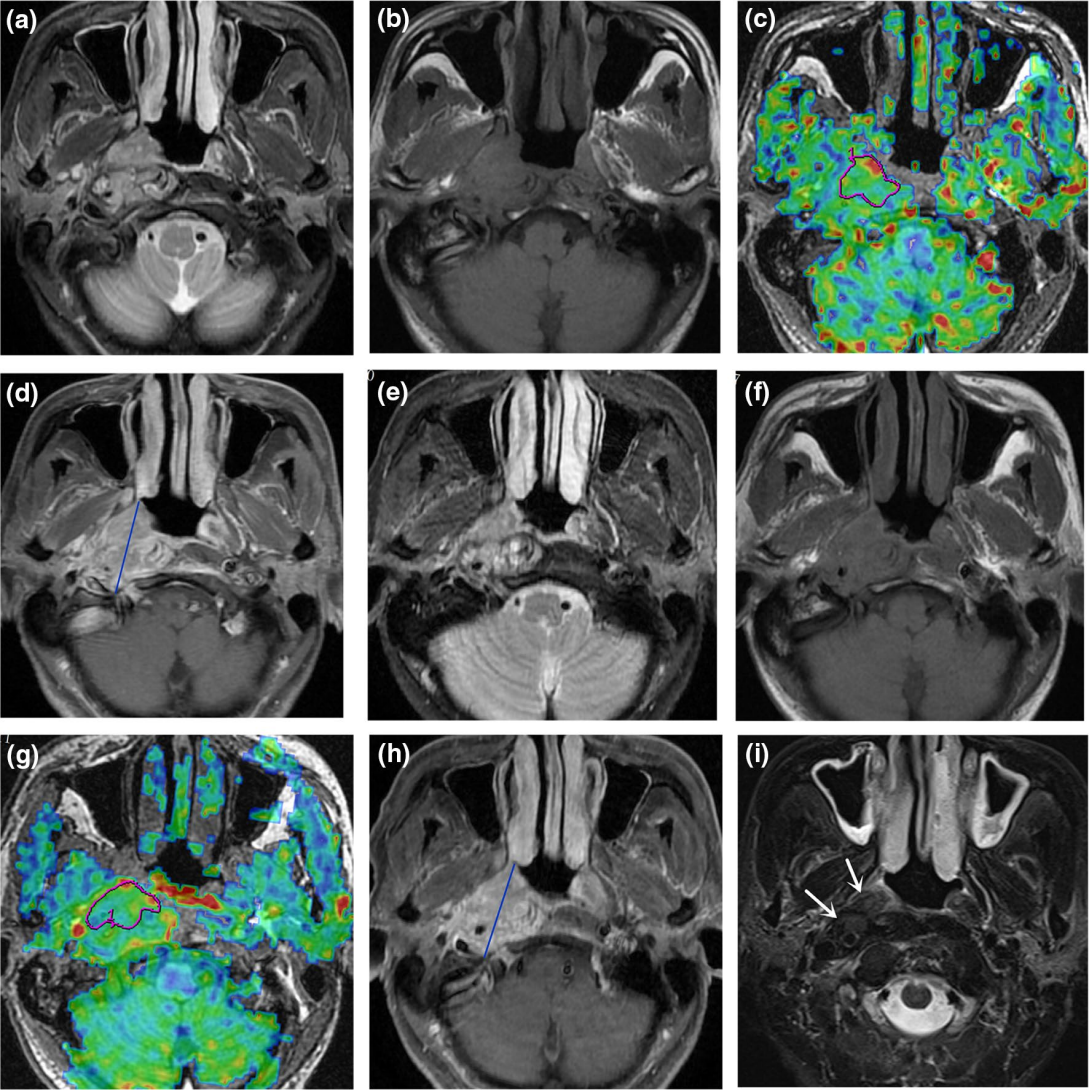
A male patient aged 57 years with stage III nasopharyngeal carcinoma (T3N2MO). Pathological examinations suggested non-keratinizing undifferentiated squamous cell carcinoma. Before treatment, T2-IDEAL and T1WI images revealed nasopharyngeal masses on the right around the right levator veli palatini, tensor veli palatini, and internal carotid artery (**a** and **b**); the ASL series integrated with BRAVO series revealed high mass perfusion (**c**) with a TBF value of 110.34 mL·100 g^−1^·min^−1^; the maximum primary tumor diameter measured on axial T1WI enhanced imaging was 37.84 mm (**d**). Re-examination after the prescribed dose reached 40 Gy suggested minor tumor shrinkage (**e** and **f**) and slightly high mass perfusion (**g**), with a TBF value of 100.32 mL·100 g^−1^·min^−1^; the maximum primary tumor diameter measured on axial T1WI enhanced imaging was 36.14 mm (**h**). Follow-up examination one month after chemoradiotherapy completion revealed a residual tumor (arrows in **i**)

**Fig. 4:**
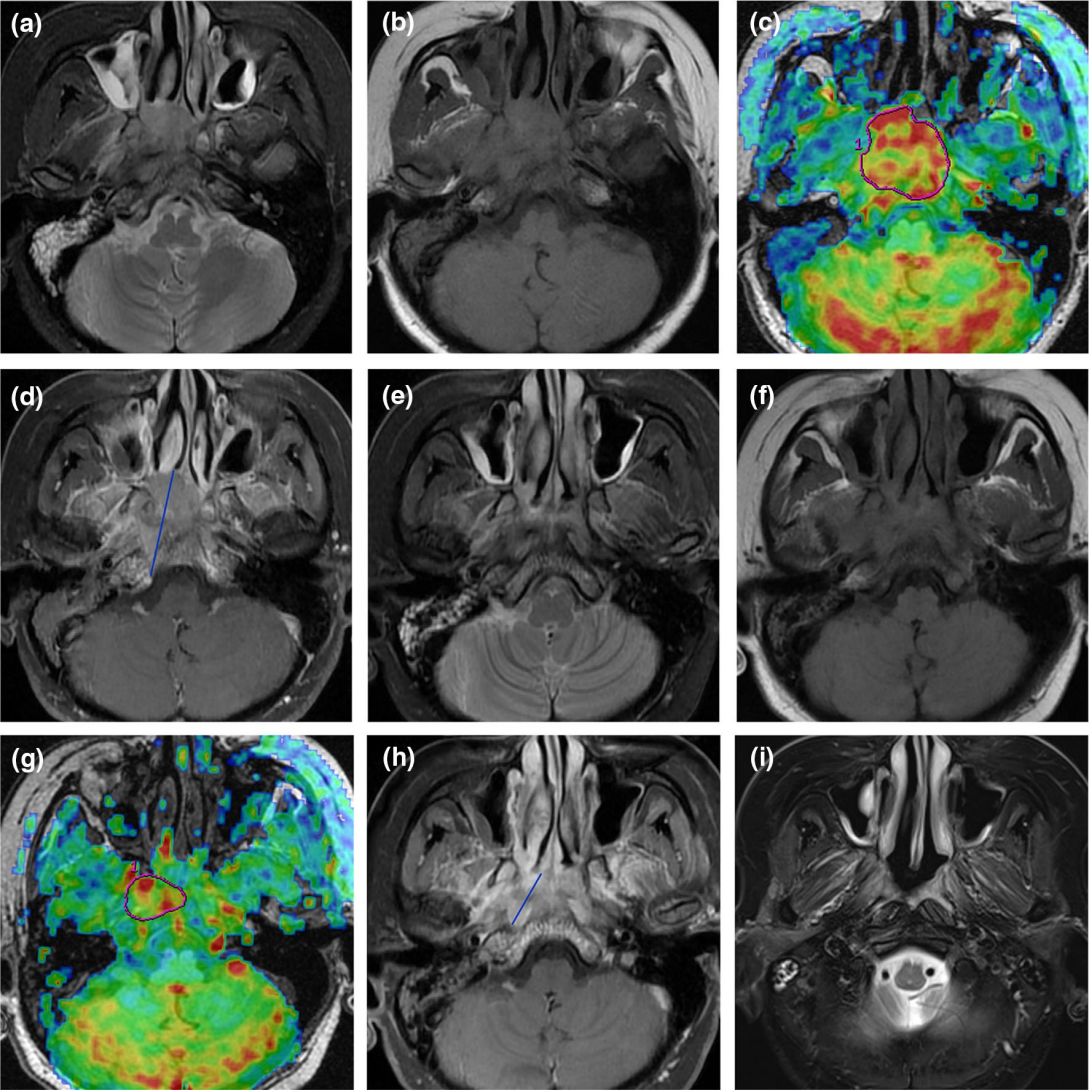
A male patient aged 48 years with stage IVA nasopharyngeal carcinoma (T4N2MO). Pathological examinations suggested non-keratinizing undifferentiated squamous cell carcinoma. Before treatment, T2-IDEAL and T1WI images revealed nasopharyngeal masses on the right invading the sphenoid sinus (**a** and **b**); the ASL series integrated with BRAVO series revealed high mass perfusion (**c**) with a TBF value of 170.67 mL·100 g^−1^·min^−1^; the maximum primary tumor diameter measured on axial T1WI enhanced imaging was 45.92 mm (**d**). Re-examinations after the prescribed dose reached 40 Gy revealed notable tumor shrinkage (**e** and **f**) and reduced tumor perfusion (**g**) with a TBF value of 107.73 mL·100 g^−1^·min.^−1^; the maximum primary tumor diameter measured on axial T1WI enhanced imaging was 24.34 mm (**h**). Follow-up examination one month after chemoradiotherapy completion revealed no residual tumor (**i**)

**Fig. 5:**
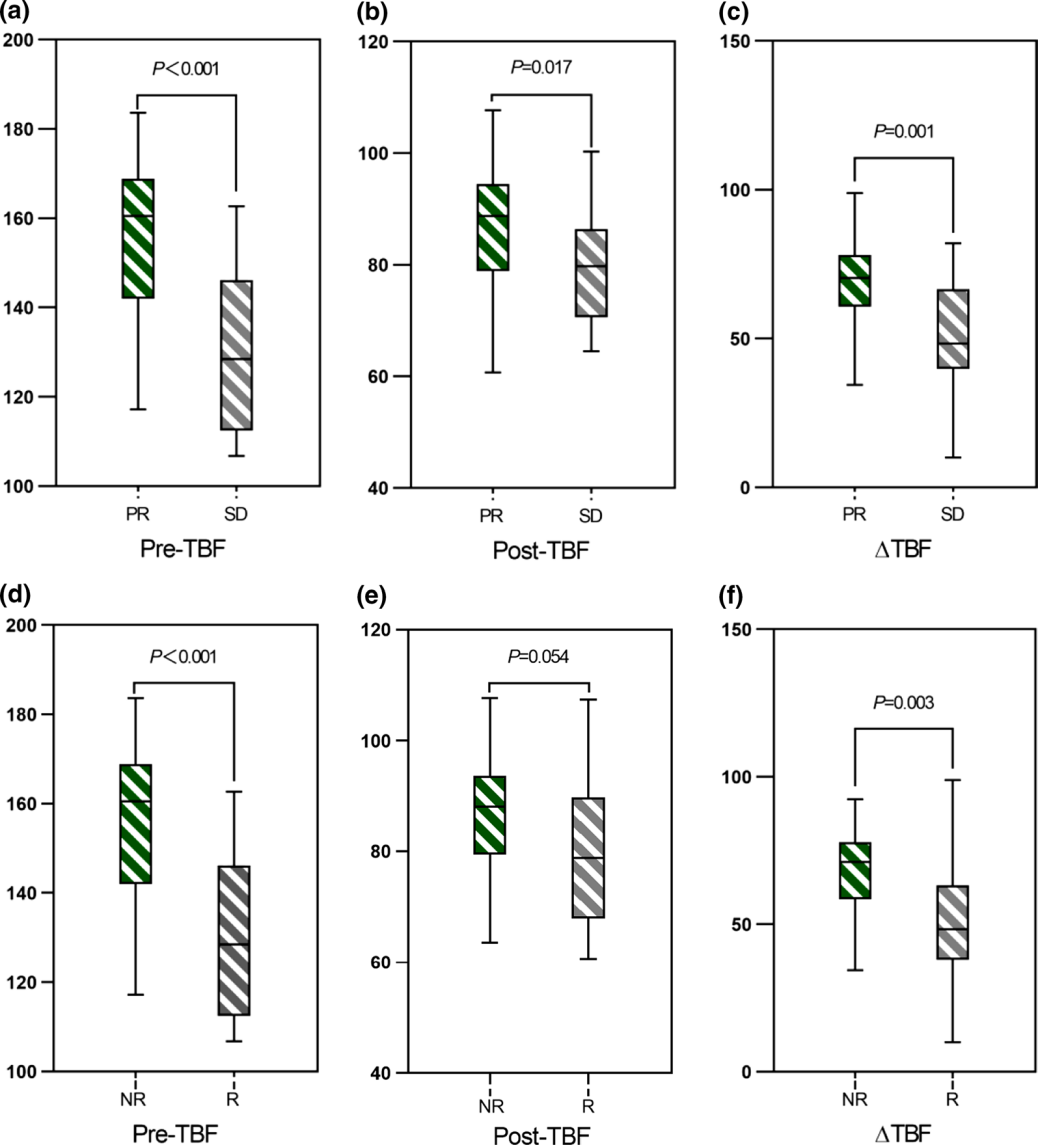
Box plots demonstrated the inter-group differences in ASL-derived parameters between the PR and SD groups, as well as between the residual and the non-residual groups. The horizontal line denotes the median values of the parameters (the 50th percentile). The top and bottom of the box denote the 25th and the 75th percentile, respectively. As can be observed from the box plots, Pre-TBF and ΔTBF can distinguish between the PR and the SD group, as well as between the residual and the non-residual group (**a**, **c**, **d**, and **f**), whereas the Post-TBF of the residual group showed an overlap with the non-residual group (**b** and **e**). Pre-TBF: pre-treatment tumor blood flow; Post-TBF: post-treatment tumor blood flow; ΔTBF: changes in tumor blood flow; PR: the partial response group; SD: the stable disease group; R: the residual group; NR: the non-residual group

**Fig. 6:**
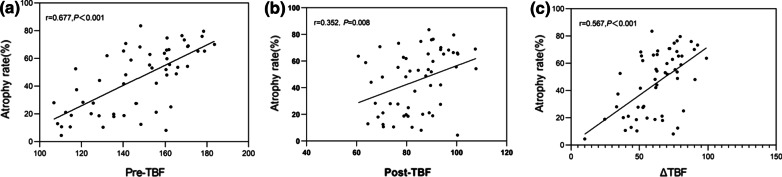
Scatterplots showing correlations between ASL-derived parameters and the tumor atrophy rate. Pre-TBF: pre-treatment tumor blood flow; Post-TBF: post-treatment tumor blood flow; ΔTBF: changes in tumor blood flow

The 55 patients with NPC demonstrated greater tumor perfusion than that of surrounding tissues on the ASL pseudocolored maps (Figs. 3c and 4c). Pre-TBF was 146.70 ± 21.39 mL·100 g^−1^·min^−1^ and Post-TBF was 84.18 ± 11.56 mL·100 g^−1^·min^−1^, the paired sample t-test indicated significantly reduced tumor perfusion after treatment (*p* < 0.001; Fig. 3g and 4g). For the PR group, the Pre-TBF value was 158.46 ± 15.74 mL·100 g^−1^·min^−1^, Post-TBF value was 91.01 ± 12.42 mL·100 g^−1^·min^−1^, and ΔTBF was 66.81 ± 14.20 mL·100 g^−1^·min^−1^. For the SD group, the Pre-TBF value was 129.41 ± 17.94 mL·100 g^−1^·min^−1^, Post-TBF value was 79.22 ± 9.55 mL·100 g^−1^·min^−1^, and ΔTBF was 46.69 ± 18.63 mL·100 g^−1^·min^−1^, The independent sample t-test showed significant differences between the inter-group measurements. For the residual group, the Pre-TBF value was 129.34 ± 19.67 mL·100 g^−1^·min^−1^ and ΔTBF was 50.15 ± 20.24 mL·100 g^−1^·min^−1^; for the non-residual group, the Pre-TBF value was 154.47 ± 17.32 mL·100 g^−1^·min^−1^ and ΔTBF was 68.05 ± 14.62 mL·100 g^−1^·min^−1^. The independent sample t-test showed significant differences between the inter-group measurements. There were no differences in Post-TBF between the residual group and the non-residual group (Table 4, Fig. 5). Pre-TBF and ΔTBF showed significant positive correlations with the tumor atrophy rate; the correlation coefficients were 0.677 and 0.567, respectively (Fig. 6).

**Table 4:** Differences in ASL-derived parameters between the SD and PR groups and residual and non-residual groups

Parameters	Group		*p-*value	Group		*p-*value
	SD (*n* = 19)	PR (*n* = 36)		Residual (*n* = 17)	No-residual (*n* = 38)	
Pre-TBF	129.87 ± 18.35	155.59 ± 17.26	< 0.001	129.34 ± 19.67	154.47 ± 17.32	< 0.001
Post-TBF	79.36 ± 9.79	86.73 ± 11.73	0.017	79.19 ± 13.04	86.42 ± 10.25	0.054
ΔTBF	50.50 ± 18.43	68.86 ± 15.05	0.01	50.15 ± 20.24	68.05 ± 14.62	0.003
Atrophy rate%	17.66 ± 6.99	60.02 ± 12.09	< 0.001	29.59 ± 20.28	52.46 ± 20.87	0.001

*Pre-TBF* pre-treatment tumor blood flow; *Post-TBF* post-treatment tumor blood flow; *ΔTBF* changes in tumor blood flow; *SD* the stable disease group; *PR* the partial response group; TBF in units of mL·100 g^−1^·min.^−1^

### Value of ASL-derived parameters in predicting early therapy response in NPC

**Fig. 7:**
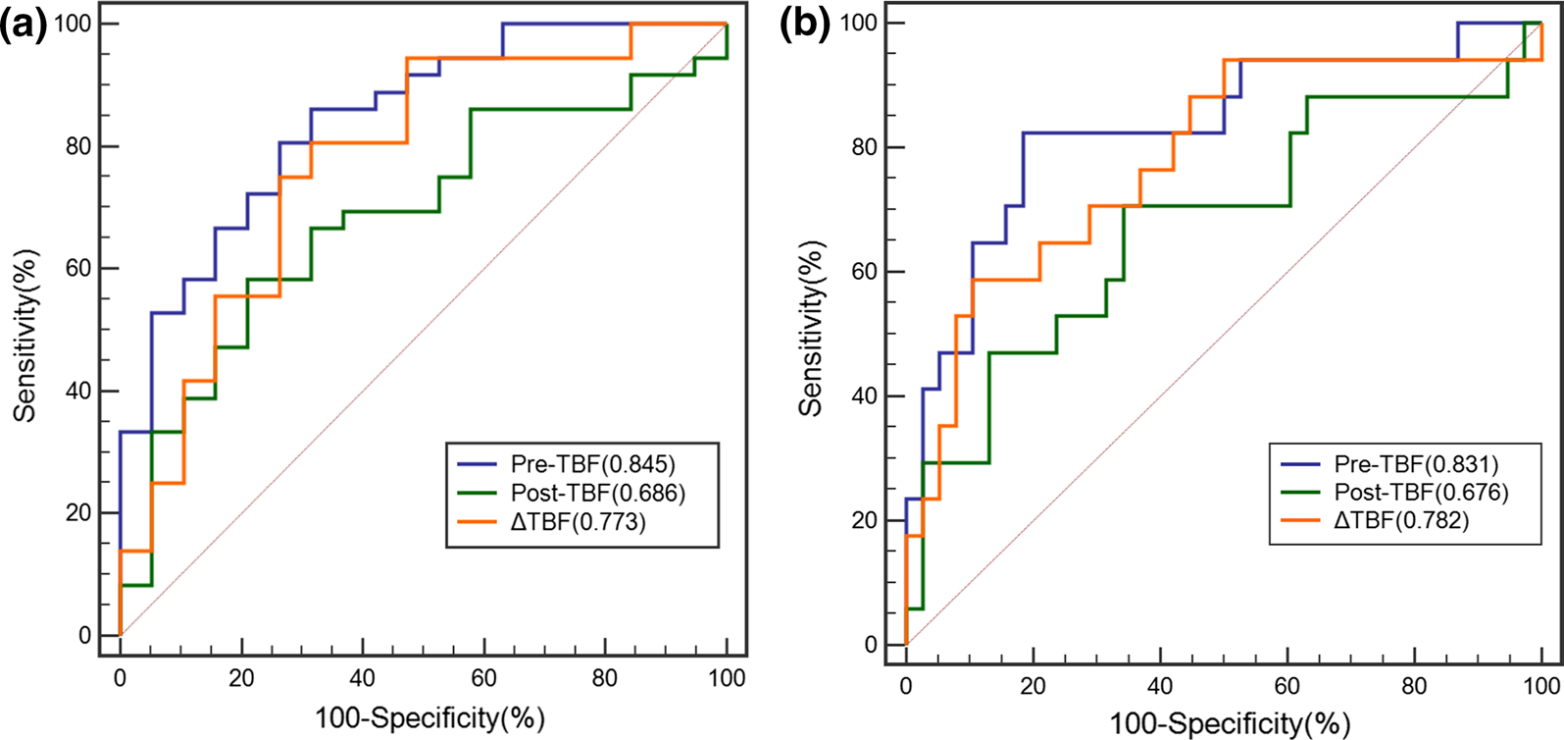
The diagnostic accuracies of ASL-derived parameters in predicting the sensitivity to chemoradiotherapy and post-treatment residual tumors. The receiver operating characteristic (ROC) curve (**a**) represents the diagnostic efficacies of Pre-TBF (blue), Post-TBF (green), and ΔTBF (orange) in predicting sensitivity to chemoradiotherapy; ROC curve (**b**) represents the diagnostic efficacies of Pre-TBF (blue), Post-TBF (green), and ΔTBF (orange) in predicting post-treatment residual tumors. Pre-TBF: pre-treatment tumor blood flow, Post-TBF: post-treatment tumor blood flow, ΔTBF: changes in tumor blood flow

The ROC curve was plotted to analyze the diagnostic efficacies of Pre-TBF, Post-TBF, and ΔTBF in predicting the efficacy of chemoradiotherapy for NPC and residual tumors. Pre-TBF was the most effective parameter at predicting sensitivity to chemoradiotherapy for NPC, with an AUC of 0.845, sensitivity of 0.861, specificity of 0.684, and threshold value of 137.07 mL·100 g^−1^·min^−1^; ΔTBF was the second most effective parameter at predicting sensitivity to chemoradiotherapy for NPC, with an AUC of 0.773, sensitivity of 0.806, specificity of 0.684, and threshold value of 57.52 mL·100 g^−1^·min^−1^. Pre-TBF was the most effective parameter at predicting post-treatment residual tumors, with an AUC of 0.831, sensitivity of 0.816, specificity of 0.824, and threshold value of 140.87 mL·100 g^−1^·min^−1^; ΔTBF was the second most effective parameter at predicting post-treatment residual tumors, with AUC = 0.782, sensitivity of 0.895, specificity of 0.588, and threshold value of 50.94 mL·100 g^−1^·min^−1^ (Table 5 and Fig. 7).

**Table 5:** Diagnostic efficacy of ASL-derived parameters in predicting PR and SD and residual and non-residual tumors

Parameters	Cutoff value (mL·100 g^−1^·min^−1^)	AUC (95% CI)	Sensitivity	Specificity
Pre-TBF^a^	137.07	0.845 (0.739,0.951)	0.861	0.684
Post-TBF^a^	87.03^1^	0.686 (0.541,0.830)	0.583	0.789
ΔTBF^a^	57.52	0.773 (0.638,0.909)	0.806	0.684
Pre-TBF^b^	140.87	0.831 (0.706,0.957)	0.816	0.824
Post-TBF^b^	82.71	0.676 (0.511,0.842)	0.658	0.706
ΔTBF^b^	50.94	0.782 (0.642,0.922)	0.895	0.588

*PR* the partial response group; *SD* the stable disease group; *Pre-TBF* pre-treatment tumor blood flow; *Post-TBF* post-treatment tumor blood flow; *ΔTBF* changes in tumor blood flow; *AUC* area under the curve; *CI* confidence interval

^a^the PR group and the SD group

^b^the residual and non-residual tumors

## Discussion

NPC is highly sensitive to radiotherapy, and radiotherapy combined with concurrent chemotherapy can improve therapeutic efficacy, enable early prediction of tumor response to chemotherapy and radiotherapy, and facilitate decisions on the optimal treatment regimen for patients with locoregionally advanced tumors. However, there is no established effective and convenient method for predicting tumor sensitivity to chemoradiotherapy in clinical practice. Hence, this study aimed to determine whether noninvasive ASL techniques reflecting tumor angiogenesis can offer reliable noninvasive imaging-based indicators to predict the early efficacy of chemoradiotherapy for patients with NPC.

NPC tissues are dependent on blood vessels, and ASL is correlated with angiogenesis markers, including microvessel density and vascular endothelial growth factor (VEFG) [22, 23]. During the course of antiangiogenic therapy, the importance of measuring TBF values is also emphasized [24], and previous studies have shown that ASL can well reflect the level of NPC perfusion [11, 17]. ASL imaging can yield images with good signal-to-noise ratio and image uniformity [25, 26], and previous studies on the kidneys [15] and bone marrow [16] indicated good reproducibility of TBF parameters. In this study, we discussed the stability of ASL data in the evaluation of chemoradiotherapy for NPC, and the study results suggested good intra-observer and interobserver agreement on measurements of TBF-quantitative parameters (ICC between 0.894 and 0.943). PLD serves as an important parameter of ASL; excessively long or short PLDs can affect tumor perfusion parameters and the image signal-to-noise ratio [27, 28]. Appropriate PLD settings are of great significance for data measurements. In previous studies on head and neck cancer and NPC, the PLD was set to different values, including 1025 ms, 1525 ms, and 2025 ms, for ASL [11, 17]. In this study, we referred to previous studies and set the PLD to 2025 ms. The obtained images provided good image contrast and measurement reproducibility, but a consensus is yet to be reached on the optimal PLD setting for individual patients.

The results of this study showed significantly higher pre-treatment TBF values of tumor tissues than post-treatment TBF values, which indicated that radiation and chemotherapeutic drugs can induce cell damage, cell necrosis, and a reduced tumor tissue perfusion. These study results were consistent with Fujima et al.’s [29], who in an ALS-based study on head and neck cancer reported significantly higher TBF values of tumor tissues before non-surgical treatment than post-treatment. Previous studies [9, 10] on the application of DCE-MRI to evaluating NPC response to chemoradiotherapy suggested higher K^trans^ (volume transfer constant) and ΔK^trans^ for the PR group than for the SD group. When analyzing the ASL and DCE of primary NPC, Lin et al. [11] found that the correlation coefficient of TBF and K^trans^ can be up to 0.688 and speculated higher TBF values in the PR group. Our study confirmed the differences in TBF values between the PR and SD groups, with higher Pre-TBF values, Post-TBF values, and ΔTBF values in the PR group. The area under the ROC curve representing the diagnostic efficacy of Pre-TBF in predicting the sensitivity of NPC to chemoradiotherapy can reach 0.845. Compared with the residual group, the non-residual group also exhibited higher Pre-TBF values and ΔTBF values. The area under the ROC curve representing the diagnostic efficacy of Pre-TBF in predicting residual tumors after chemoradiotherapy for NPC can reach 0.831. The results of this study suggest higher intra-tumoral vessel density and a higher tumor perfusion in the PR and the non-residual groups. High tumor perfusion indicates a higher sensitivity to chemoradiotherapy and a higher probability of complete tumor atrophy after treatment completion, whereas micronecrosis and cystic changes in hypoperfused tumor tissues can lead to hypoxia and acidosis, which promotes the transformation of tumor cells into subtypes more resistant to chemotherapy and radiotherapy and leads to insensitivity to chemoradiotherapy [30, 31] and a higher incidence of residual tumors at the end of treatment.

Liang et al. [32] and Ni et al. [33] found that, as an independent predictor of long-term prognosis for patients with NPC, the tumor atrophy rate is partially capable of predicting the 5-year overall survival rate and distant metastasis-free survival rates. Patients with poor atrophy should receive timely intensive therapy at the end of radiotherapy. The results of this study demonstrated a significant correlation between TBF parameters and the tumor atrophy rate, with the highest correlation between Pre-TBF values and tumor atrophy rate (correlation coefficient: 0.677). Based on this finding, it is plausible to suggest that the Pre-TBF values of nasopharyngeal tumor masses can serve as a useful reference for clinicians in the prediction of tumor atrophy rate and prognosis during patient treatment.

However, DCE techniques require the administration of exogenous Gadolinium-based contrast agents, which may cause allergy, brain deposition of gadolinium, and nephrogenic fibrosis [34]. Hence, the severe limitations and drawbacks of DCE-MRI are highlighted when the patient has allergies or renal insufficiency or requires constant monitoring of therapeutic efficacy. A study by Xiao-ping. et al. [12] found that IVIM derived indicators of perfusion, including D* (pseudo-diffusion coefficient) and *f* (perfusion fraction), were less effective at predicting the early efficacy of chemoradiotherapy for NPC than diffusion-related parameters. However, Xiao et al. [13] found that *f* was more sensitive in the early course of the treatment. This was because the indicators of perfusion in the IVIM DW-MRI model were affected by the TE, T2 relaxation time, and choice of b values [35], and data reproducibility was poor [36]. DKI and single-index DWI can be used to predict the early efficacy of chemoradiotherapy for NPC [7–9]; however, diffusion images are prone to geometric image distortions, magnetic susceptibility artifacts, and blurring due to the complex anatomy of nasopharyngeal structures, including air gaps, bones, soft tissues, and other components [37]. In contrast, ASL has good prospects in clinical application due to its advantages including no need for exogenous contrast agents, simple operation, and good image quality.

This study still has its limitations. First, the ASL series was acquired using only one PLD value, and comparisons of TBF values acquired at other PLD values could not be made. Therefore, an ASL series need to be acquired at multiple PLD values for verification in future studies. Second, this study was retrospective and involved a small sample, and factors including pre-treatment tumor staging and MPTD were not included in the analysis of sensitivity to chemoradiotherapy. Therefore, the sample size needs to be increased, and multivariate analysis needs to be performed to improve accuracy in further studies. Third, the follow-up period for this study was not extended to ≥ 3 months after treatment completion. Longer follow-up is needed to determine the potential value of ASL in predicting long-term efficacy.

## Conclusion

Several ASL-derived TBF parameters provide a noninvasive approach for assessing tumor blood perfusion and the early efficacy of chemoradiotherapy for NPC. They are highly correlated with the tumor atrophy rate. Hence, these TBF parameters are of good clinical significance and have prospects for clinical application since they can provide an effective reference for the development of a clinical treatment regimen and the prediction of patient prognosis.

## Data Availability

The data that support the findings of this study are available from Q.Y. Restrictions apply to the availability of these data, which were used under license for the current study, and therefore are not publicly available. Data are, however, available from the authors upon reasonable request and with permission from Q.Y.

## Abbreviations

ASL: Arterial spin labeling
AUC: Area under the receiver operating characteristic curve
BED: Biologically effective dose
DCE: Dynamic contrast-enhanced
DKI: Diffusion kurtosis imaging
DWI: Diffusion-weighted imaging
IVIM: Intravoxel incoherent movement
ICC: Intraclass correlation coefficient
MRI: Magnetic resonance imaging
MPTD: Maximum primary tumor diameter
NPC: Nasopharyngeal carcinoma
PR: Partial response
ROC: Receiver operating characteristic
ROI: Region of interest
SD: Stable disease
TBF: Tumor blood flow
